# Application of predicting risk of cardiovascular disease events equations on postoperative major adverse cardiac and cerebral events for patients undergoing thoracic surgery

**DOI:** 10.3389/fcvm.2026.1823917

**Published:** 2026-07-01

**Authors:** Wan-Qiu Fan, Shuang Wang, Er-Yi Zhu, Chun-Yu Yu, Yi-Kai Liu, Hong Li, Ke-Xuan Liu, Hua-Min Liu

**Affiliations:** 1 Department of Anesthesiology, Second Affiliated Hospital of Army Medical University, Chongqing, China; 2 Department of Anesthesiology, Nanfang Hospital, Southern Medical University, Guangzhou, Guangdong, China; 3 Guangdong Provincial Key Laboratory of Precision Anesthesia and Perioperative Organ Protection, Nanfang Hospital, Southern Medical University, Guangzhou, Guangdong, China

**Keywords:** major adverse cardiac and cerebral events, noncardiac thoracic surgery, PREVENT equations, risk association, sex-specific

## Abstract

**Objectives:**

The American Heart Association's Predicting Risk of Cardiovascular Disease Events (PREVENT) equations predict cardiovascular risk, but their utility in perioperative settings is unclear. This study aims to evaluate the association between PREVENT equations and postoperative cardiovascular events in thoracic surgical population.

**Methods:**

This retrospective study included 1,073 patients undergoing thoracic surgery at Nanfang Hospital, Southern Medical University, between January 2019 and October 2023. The sex-specific PREVENT risk scores for atherosclerotic cardiovascular disease (ASCVD), coronary heart disease (CHD), heart failure (HF), and total cardiovascular disease (CVD) were calculated. The study outcome was 30-day postoperative composite events of major adverse cardiac and cerebral events (MACCE: composite of angina, myocardial injury, stroke, and HF). The single event or composition combinations matched to PREVENT risk scores were also assessed: angina, HF, angina or myocardial injury (AMI), AMI or stroke (AMIS). Multivariate logistic regression was used to evaluate MACCE risk, with false discovery rate (FDR) correction for *P* values. The discriminative ability was assessed using receiver operating characteristic (ROC) curves and area under the curve (AUC).

**Results:**

The median age of included patients was 58 years old. In females, one standard-deviation (SD) increase in the ASCVD score was significantly associated with higher odds of postoperative angina (OR = 1.95, 95% CI: 1.19–3.21, FDR corrected *P* = 0.0381). CHD score was significantly associated with female angina (OR = 1.83, 95% CI: 1.14–2.95, FDR corrected *P* = 0.0354) and AMI (OR = 1.46, 95% CI: 1.05–2.03, FDR corrected *P* = 0.0386). In males, no PREVENT risk scores were significantly associated with major adverse cardiac and cerebral events (all FDR corrected *P* > 0.05). The PREVENT equations demonstrated light to moderate discrimination, with AUCs ranging from 0.65 to 0.75 in female patients.

**Conclusions:**

The PREVENT equations demonstrated preliminary associations with 30-day postoperative MACCE in non-cardiac thoracic surgical female patients, but not in male patients.

## Introduction

Perioperative major adverse cardiac and cerebral events (MACCE) are severe complications that significantly contribute to postoperative mortality and disability (1). While the incidence of perioperative MACCE in non-cardiac surgery is approximately 3% (2), it is markedly higher in thoracic surgery, reaching up to 18.2% (3–7). Although established risk models, such as the Revised Cardiac Risk Index [RCRI] (4) and Geriatric Sensitive Perioperative Cardiac Risk Index [GSCRI] (8), are widely utilized to estimate the MACCE risk (4, 8–14). However, these prediction models omitted key factors like blood lipids and kidney function, both of which are significantly associate with MACCE (15–17). Besides, the accuracy of the standard RCRI has been questioned in the specific context of thoracic surgery. This led to the development of the Thoracic Revised Cardiac Risk Index (ThRCRI), which was validated by Ferguson et al. to successfully stratify risk for major lung resection by weighting factors such as creatinine, coronary artery disease, and cerebrovascular disease. Despite these specialized refinements, current thoracic-specific models still omit key metabolic factors like blood lipids (18). Notably, these factors are core components of the newly defined Cardiovascular-Kidney-Metabolic (CKM) syndrome, a major and growing global health burden with high prevalence in both the United States and China (19–21). Moreover, CKM syndrome has been demonstrated to be associated with an increased risk of MACCE after non-cardiac surgery (22, 23). Thus, incorporating blood lipids and kidney function into risk stratification is essential.

The American College of Cardiology (ACC)/American Heart Association (AHA) developed the Predicting Risk of Cardiovascular Disease Events (PREVENT) equations. As an update of the 2013 Pooled Cohort Equations (PCEs), PREVENT equations estimate the risk of atherosclerotic cardiovascular disease (ASCVD) and Heart Failure (HF) in American adults aged 30–79 (24). These equations integrate lipids and estimated glomerular filtration rate (eGFR), creating a comprehensive prediction tool containing metabolic, renal and cardiovascular risk factors, which precisely lack in previous prediction models. Compared to the 2013 PCEs, the PREVENT equations better assess CVD risk across populations by removing race-specific coefficients from the algorithm (24, 25). This change mitigates biases from simplistic racial categorization, thereby enhancing the model's generalizability. Preliminary validation suggested that PREVENT's race-unadjusted framework could improve calibration in external cohorts without compromising discriminatory ability (26, 27).

The PREVENT equations have been externally validated in high-risk cohorts (e.g., chronic kidney disease, diabetes, statin users) (26, 28, 29), demonstrating robust performance across these patients. In addition, researchers emphasize that comprehensive validation across diverse clinical populations should be prioritized before incorporating the PREVENT equations into clinical guidelines and practice (30). Moreover, all the parameters in PREVENT equations are relevant to postoperative cardiovascular complications, we hypothesized that these equations are also applicable to surgical populations. This may be a new application field of PREVENT equations. Accordingly, we explored the association between PREVENT equations and postoperative cardiovascular events in non-cardiac thoracic surgical patients.

## Methods

### Study design and participants

**Figure 1:**
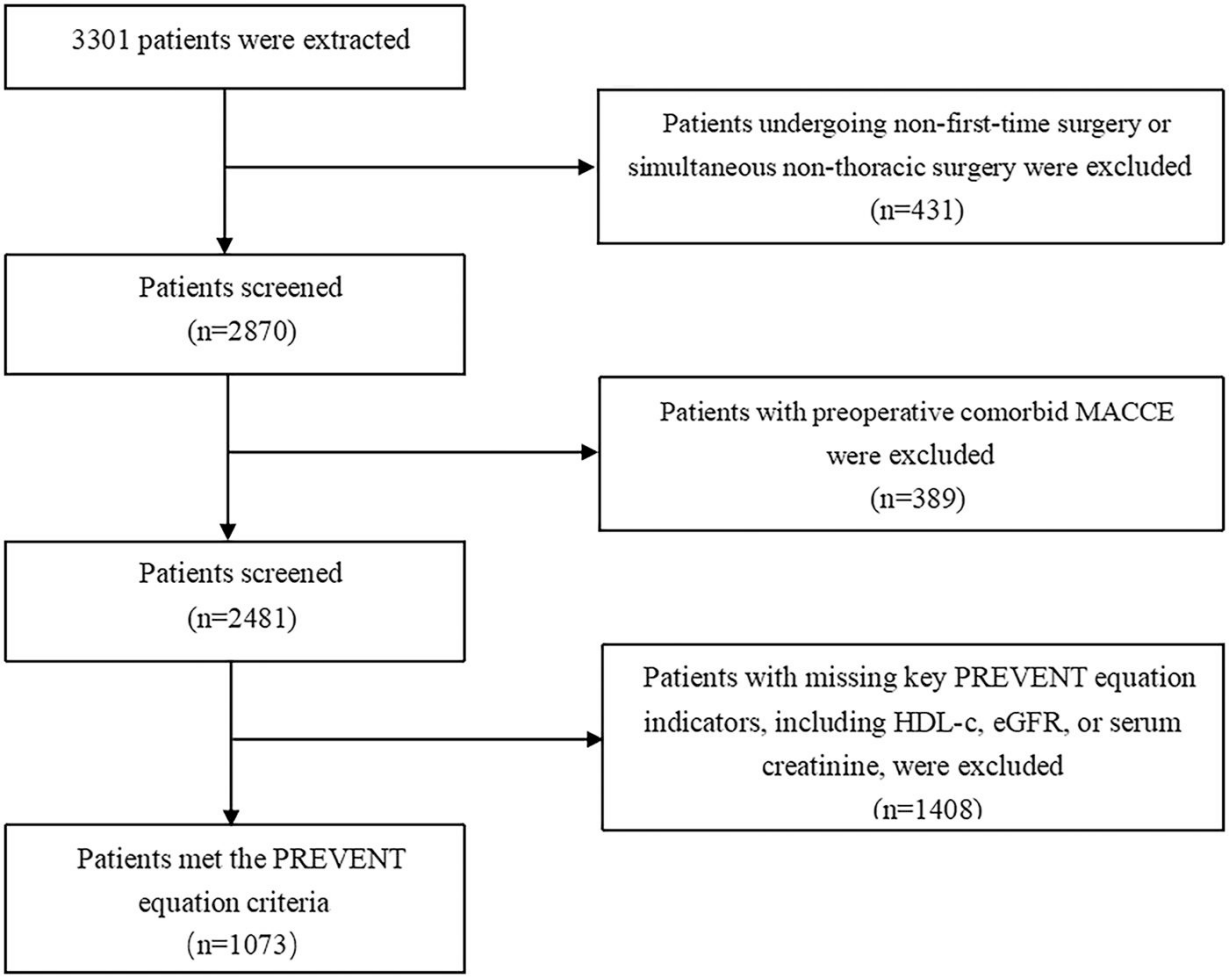
Flow-chart of patients' selection. MACCE, major adverse cardiac and cerebral events, including angina, myocardial injury stroke or heart failure; PREVENT, Predicting Risk of Cardiovascular Disease EVENTs.

We conducted a retrospective cohort study of patients who underwent non-cardiac thoracic surgery at Nanfang Hospital, Southern Medical University, between January 2019 and October 2023. The study protocol was approved by the Medical Ethics Committee of Nanfang Hospital, Southern Medical University (NFEC-2023-573), which granted a waiver of informed consent due to the retrospective design. From a Perioperative Data Warehouse of 3,301 initial patients, we excluded 431 patients who underwent repeat or concurrent non-thoracic surgeries, 389 with severe preoperative cardiovascular events, and 1,408 with missing data required for the PREVENT equations. The final analysis included 1,073 patients (Figure 1).

### PREVENT equations

We applied the four basic sex-specific models in PREVENT equations, including the total CVD, ASCVD, HF, and CHD scores, to estimate the 30-day odds of postoperative cardiovascular events. Predictors included age, smoking status, systolic blood pressure (SBP), use of anti-hypertensive medication, statin use, diabetes, high-density lipoprotein cholesterol (HDL), total cholesterol (TC), estimated glomerular filtration rate (eGFR) and body-mass index (BMI). The calculation of PREVENT score is sex-specific, with distinct formulas for males and females. Therefore, the study hypothesis was tested in males and females, respectively. Formulas details are provided in Supplementary Table S1.

### Outcomes and corresponding risk scores calculated by PREVENT equations

The study outcome was MACCE within 30 days postoperatively, defined as a composite of myocardial injury, heart failure, angina, and stroke. These events were identified using the International Classification of Diseases, 10th Revision (ICD-10) codes retrieved from electronic medical records. Specifically, angina was identified by codes starting with I20.x, myocardial infarction by I21.x and I24.x, stroke by I63.x, and heart failure by I50.x. Myocardial injury was determined by elevated troponin levels (peak ≥20 ng/L and absolute change ≥5 ng/L). Besides, to minimize ascertainment bias, we reviewed postoperative imaging examinations' reports (including transthoracic echocardiography, computed tomography angiography and non-contrast head computed tomography) and clinical consultation notes. Any description about the outcomes was defined as MACCE. This method ensured the consistency and completeness of postoperative complication identification across the entire cohort.

Outcomes were mapped to different risk score in PREVENT equations based on shared pathophysiology. Given the common atherosclerotic basis, angina odds were estimated using the CHD and ASCVD scores. HF was assessed using the HF score. Composite outcomes were also evaluated: angina and myocardial injury (AMI) with the CHD score; angina, myocardial injury, and stroke (AMIS) with the ASCVD score; and the full MACCE composite (angina, myocardial injury, stroke, or heart failure) with the total CVD score. The specific risk scores corresponding to each outcome is detailed in Table 1.

**Table 1:** Outcomes and corresponding risk scores.

**Outcomes**	**Complication (s)**	**Risk Scores**
MACCE	Angina, myocardial injury, stroke, HF	Total CVD score
HF	HF	HF score
Angina	Angina	CHD score
Angina	Angina	ASCVD score
AMI	Angina, myocardial injury	CHD score
AMIS	Angina, myocardial injury, stroke	ASCVD score

AMI, angina and myocardial injury; AMIS, angina, myocardial injury and stroke; ASCVD, atherosclerotic cardiovascular disease; CHD, coronary heart disease; CVD, cardiovascular disease; HF, heart failure; MACCE, major adverse cardiac and cerebral events, including angina, myocardial injury, stroke, or heart failure.

### Covariates

Covariates were selected based on clinical relevance and literature review. They included a range of preoperative variables: laboratory values [hemoglobin, platelets, white blood cell count, absolute neutrophil count, neutrophil (%), absolute lymphocyte count, lymphocyte (%), C-reactive protein (CRP), procalcitonin (PCT), triglycerides (TG), low-density lipoprotein (LDL), albumin, prothrombin time (PT), activated partial thromboplastin time (APTT), glycated hemoglobin (HbA1c), glucose, N-terminal pro-B-type natriuretic peptide (NT-proBNP)], clinical conditions (atrial arrhythmia, arrhythmia), and other factors [aspirin use, current drinking, American Society of Anesthesiologists (ASA) Physical Status].

### Statistical analysis

Statistical analyses were performed using R version 4.4.1 (R Foundation for Statistical Computing, Vienna, Austria). Missing data were imputed using the random forest algorithm (use the missRanger R package). We analyzed the patterns of missing values using a heatmap to verify the random missing pattern (Supplementary Figure S1, Table S2).

Continuous variables, which were identified as non-normally distributed by the Kolmogorov–Smirnov test, are presented as median (interquartile range, IQR) and compared using the Mann–Whitney *U*-test. Categorical variables are expressed as number (percentage) and compared using the Pearson chi-square test or Fisher's exact test, as appropriate.

Restricted cubic spline analysis was used to examine the functional form (nonlinear or linear) of the associations between the PREVENT risk scores and cardiovascular events. The risk scores were standardized (mean = 0, SD = 1) for model stabilization. We used univariate logistic regression to assess associations between PREVENT scores and various cardiovascular events, followed by multivariable logistic regression to adjust for potential confounders. Covariates for adjustment were selected using least absolute shrinkage and selection operator (LASSO) regression (Supplementary Figure S2). Adjusted covariates in females included ASA, arrhythmia, aspirin, current-drink, hemoglobin, neutrophil percentage, CRP, PT, glucose, and NT-proBNP, while in males included atrial arrhythmia, aspirin, current-drink, hemoglobin, CRP, and TG. According to the usage instructions of the PREVENT equations, we tested our hypothesis separately in males and females. Thus, six comparisons were performed in males and females, respectively. To account for multiple testing and control the false discovery rate (FDR), we applied the Benjamini–Hochberg (BH) procedure. The Q threshold of FDR correction was set as 0.05. Adjusted *P*-values were calculated as: [p(i) × m]/i, where the p(i) was the ordered unadjusted *P* values, and m = 6 represents the total number of comparisons. Associations with adjusted *P*-values <0.05 were considered statistically significant.

Model performance was evaluated using receiver operating characteristic (ROC) curves, and optimal cut-off values were determined. We compared the PREVENT score with commonly used clinical perioperative risk assessment tools, specifically the Revised Cardiac Risk Index (RCRI) and the Thoracic Revised Cardiac Risk Index (ThRCRI). Model discrimination was assessed using the area under the receiver operating characteristic curve (AUC). Additionally, we calculated the Integrated Discrimination Improvement (IDI) and Net Reclassification Index (NRI) to evaluate the incremental predictive value and reclassification performance of the PREVENT equations compared to RCRI and ThRCRI. A sensitivity analysis was conducted using the non-imputed dataset. Another sensitivity analysis was conducted by imputing the input variables of the PREVENT equations.

Following the rule of thumb of 10 events per variable (10 EPV) (31), the number of MACCE in this study exceeded the reliable estimate for covariates in a multivariate model. A two-sided *P*-value <0.05 was considered statistically significant.

## Results

### Baseline characteristics

Baseline characteristics of the female and male cohorts are shown in Table 2. Regarding PREVENT equation factors, females had significantly higher levels of TC, HDL-C, and eGFR, but lower age and SBP compared to males (all *P* < 0.05). The proportion of current smokers was higher in males (*P* < 0.05). Other characteristics that differed significantly between groups included hemoglobin, CRP, NT-proBNP, current drinking, atrial arrhythmia, and arrhythmia (all *P* < 0.05).

**Table 2:** Characteristics of the patients.

Characteristics	Total (*n* = 1,073)	Female (*n* = 430)	Male (*n* = 643)	*P* value
Age (years), Median (IQR)	58 (51, 65)	58 (50, 65)	58 (52, 66)	0.05
BMI ((kg/m^2^), Median (IQR)	23.4 (21.4, 25.6)	23.1 (21.2, 25.2)	23.5 (21.6, 25.8)	0.05
SBP (mmHg), Median (IQR)	130 (121, 140)	128 (120, 138)	130 (122, 140)	0.05
TC (mg/dL), Median (IQR)	188 (162, 218)	196 (166, 226)	185 (160, 213)	<0.001
HDL-C (mg/dL), Median (IQR)	45 (39, 53)	49 (42, 58)	42 (37, 49)	<0.001
eGFR, Median (IQR)	82.8 (65.6, 97.2)	97.7 (85.8, 105.7)	71.6 (60.5, 85.1)	<0.001
Statin use, *n* (%)	255 (23.8)	107 (24.8)	148 (23.0)	0.48
Hypertension medication (*n*, %)	368 (34.3)	149 (34.6)	219 (34.0)	0.84
Diabetes, *n* (%)	199 (18.5)	72 (16.7)	127 (19.7)	0.21
Current smokers, *n* (%)	274 (25.5)	7 (1.6)	267 (41.5)	<0.001
Hemoglobin (g/L), Median (IQR)	131 (120, 142)	124 (116, 132)	138 (126, 147)	<0.001
Neutrophil (%), Median (IQR)	58.0 (51.9, 64.8)	57.5 (50.9, 64.7)	58.40 (52.95, 64.95)	0.05
CRP (mg/L), Median (IQR)	1.5 (0.7, 4.1)	1.4 (0.7, 3.8)	1.7 (0.7, 4.3)	0.04
Triglyceride (mg/dL), Median (IQR)	121 (89, 180)	125 (92, 177)	118 (86, 181)	0.19
PT (seconds), Median (IQR)	17.7 (17.0, 18.4)	17.7 (17.0, 18.4)	17.7 (17.0, 18.4)	0.87
Glucose (mmol/L), Median (IQR)	5.1 (4.7, 5.6)	5.0 (4.7, 5.5)	5.1 (4.6, 5.7)	0.27
NT-proBNP (pg/mL), Median (IQR)	39.5 (21.5, 73.8)	42.0 (24.2, 77.4)	38.5 (20.7, 71.1)	0.04
Current Drink, *n* (%)	344 (32.1)	52 (12.1)	292 (45.4)	<0.001
ASA, *n* (%)				0.44
Ⅰ	41 (3.8)	16 (3.7)	25 (3.9)	
II	877 (81.7)	359 (83.5)	518 (80.6)	
≥III	155 (14.5)	55 (12.8)	100 (15.6)	
Atrial arrhythmia, *n* (%)	36 (3.4)	8 (1.86)	28 (4.4)	0.03
Arrhythmia, *n* (%)	38 (3.5)	9 (2.1)	29 (4.5)	0.04
Aspirin medication, *n* (%)	69 (6.4)	31 (7.2)	38 (5.9)	0.40

ASA, American society of Anesthesiologists physical status; BMI, body mass index; CRP, C-reactive protein; eGFR, estimated glomerular filtration rate; HDL-C, high-density lipoprotein cholesterol; IQR, interquartile range; PT, prothrombin time; SBP: systolic blood pressure; TC, total cholesterol.

### Restricted cubic spline analysis of PREVENT equations and MACCE

**Figure 2:**
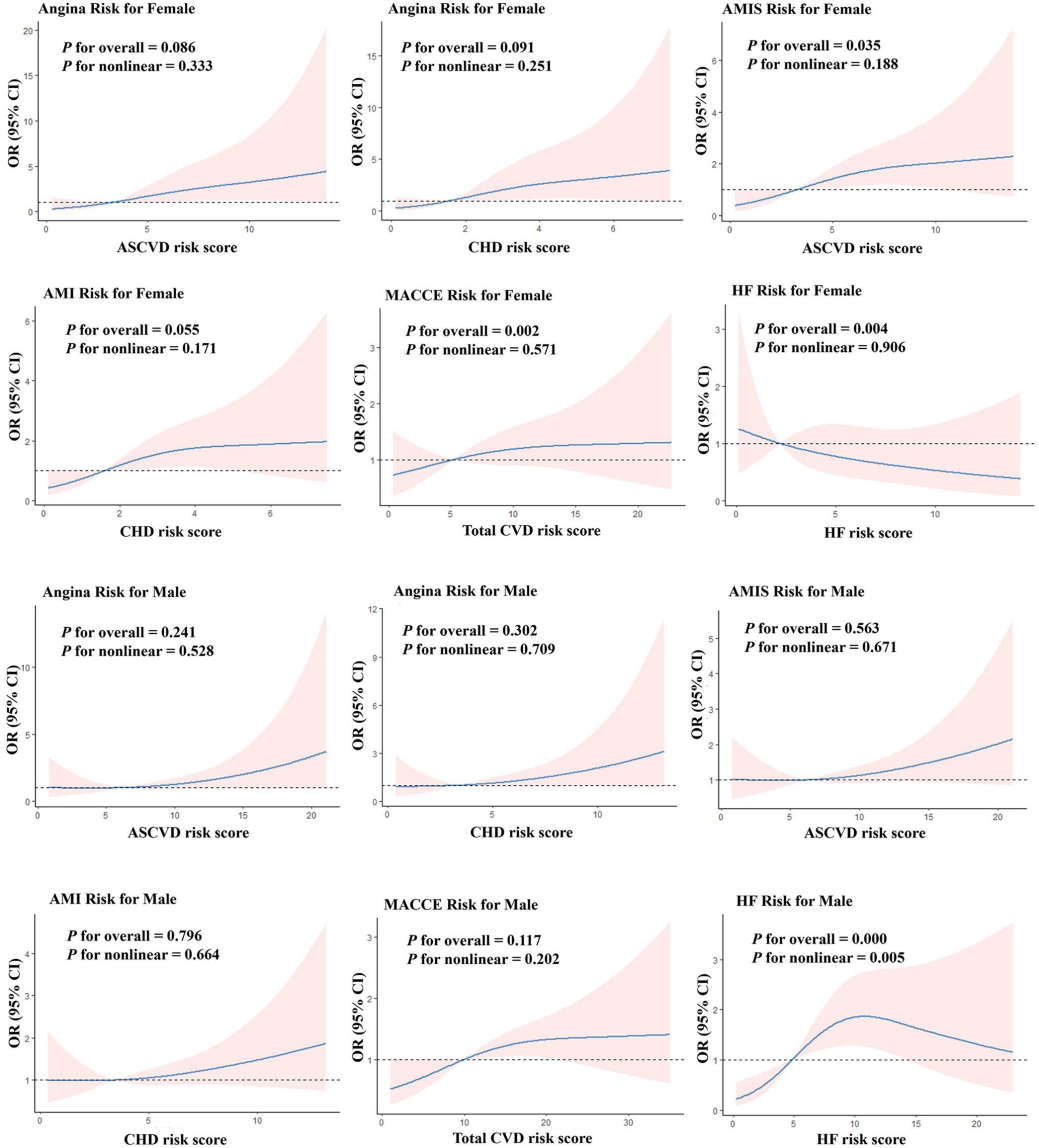
Restricted cubic spline curve for the association between PREVENT equations and major adverse cardiac and cerebral events. AMI, angina and myocardial injury; AMIS, angina, myocardial injury and stroke; ASCVD, atherosclerotic cardiovascular disease; CHD, coronary heart disease; CVD, cardiovascular disease; HF, heart failure; MACCE, major adverse cardiac and cerebral events, including angina, myocardial injury, stroke, or heart failure.

In females, restricted cubic spline analyses revealed positive linear associations between several PREVENT scores and cardiovascular events, including: angina with the ASCVD score, angina with the CHD score, AMI with the CHD score, AMIS with the ASCVD score, and MACCE with the total CVD score. Conversely, the HF score demonstrated a negative correlation with the odds of HF (Figure 2).

In males, positive correlations were observed for the same set of events and scores as in females. Additionally, a significant nonlinear relationship was identified between the HF score and the odds of HF (*P* for nonlinearity = 0.005), with an inflection point at an HF score of 10.998 (Figure 2).

### Logistic regression analyses of PREVENT equations and MACCE

**Figure 3:**
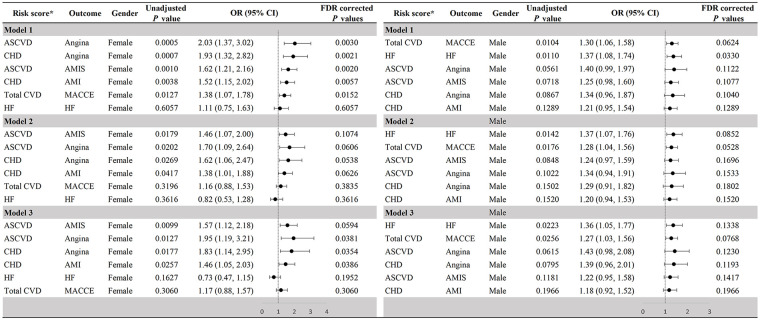
Association of PREVENT equations with major adverse cardiac and cerebral events. *Risk scores were Z-transformed. Model 1: unadjusted. Model 2 for females: adjusted for ASA classification, arrhythmia, aspirin, current-drink; Model 2 for males: adjusted for atrial arrhythmia, aspirin, current-drink; Model 3 for females: adjusted for ASA classification, arrhythmia, aspirin, current-drink, hemoglobin, neutrophil (%), C-reactive protein (CRP), PT, glucose, and NT-proBNP; Model 3 for males: adjusted for atrial arrhythmia, aspirin, current-drink, hemoglobin, CRP, and triglyceride. AMI, angina and myocardial injury; AMIS, angina, myocardial injury and stroke; ASCVD, atherosclerotic cardiovascular disease; CHD, coronary heart disease; CVD, cardiovascular disease; HF, heart failure; MACCE, major adverse cardiac and cerebral events, including angina, myocardial injury, stroke, or heart failure.

Females who experienced angina, AMI, AMIS, or MACCE had higher corresponding PREVENT scores (Supplementary Figure S3). As shown in Figure 3, univariate analyses indicated that the odds ratios (ORs) and 95% confidence intervals (CIs) of angina, AMI, AMIS, and MACCE were increased with corresponding PREVENT scores, and all the FDR corrected *P* values were statistically significant. After adjusting for potential confounders, the FDR corrected *P* values of angina and AMI were still significant (both FDR corrected *P* values <0.05), the FDR corrected *P* value of AMIS changed borderline significant (FDR corrected *P* value = 0.0594), whereas the increase odds of MACCE lost significance (FDR corrected *P* value = 0.3060).

Among males, those with MACCE or HF had higher corresponding PREVENT scores (Supplementary Figure S3). Univariate analysis showed that the odds of HF was increased with HF score (OR: 1.37; 95% CI: 1.08–1.74, FDR corrected *P* value: 0.0330). However, this association did not remain significant after adjusting for potential confounders (FDR corrected *P* value: 0.1338, Figure 3). No statistically significant associations were observed for other PREVENT equations and their corresponding outcomes (all FDR corrected *P* values >0.05).

Given the nonlinear association identified between HF and the HF score, we separately performed logistic regression analyses stratified by the inflection point (10.998), without FDR correction. HF score below the inflection point was significantly associated with increased odds of HF in both univariate [OR (95% CI): 2.55 (1.30–5.00)] and multivariable analyses [OR (95% CI): 2.55 (1.23–5.28)]. In contrast, no significant association was observed for HF score above 10.998 [adjusted OR (95% CI): 0.57 (0.26, 1.28)] (Supplementary Table S3).

### Sensitivity analyses for the associations between PREVENT equations and cardiovascular events

We conducted a sensitivity analysis on the dataset without imputation (*n* = 785). The results were similar to the primary results (Supplementaty Figure S4, Table S4).

Baseline characteristics between the included patients and the patients with missing data of PREVENT equations input variables (*n* = 1,408) are presented in Supplementary Table S5. Most characteristics were significantly different. However, no significant differences were observed between the two groups in the incidence of outcomes within 30 days postoperatively (*P* > 0.05). Supplementary Table S6 provides details of missing data for PREVENT equations input variables among the 1,408 excluded patients. Notably, sensitivity analyses incorporating imputed data from these excluded patients demonstrated different findings from our primary results. Each PREVENT equations were significantly associated with corresponding outcomes, except for HF score in females and CHD score in males (Supplementary Figure S5, Table S7).

### Discrimination of the PREVENT equations on MACCE

**Figure 4:**
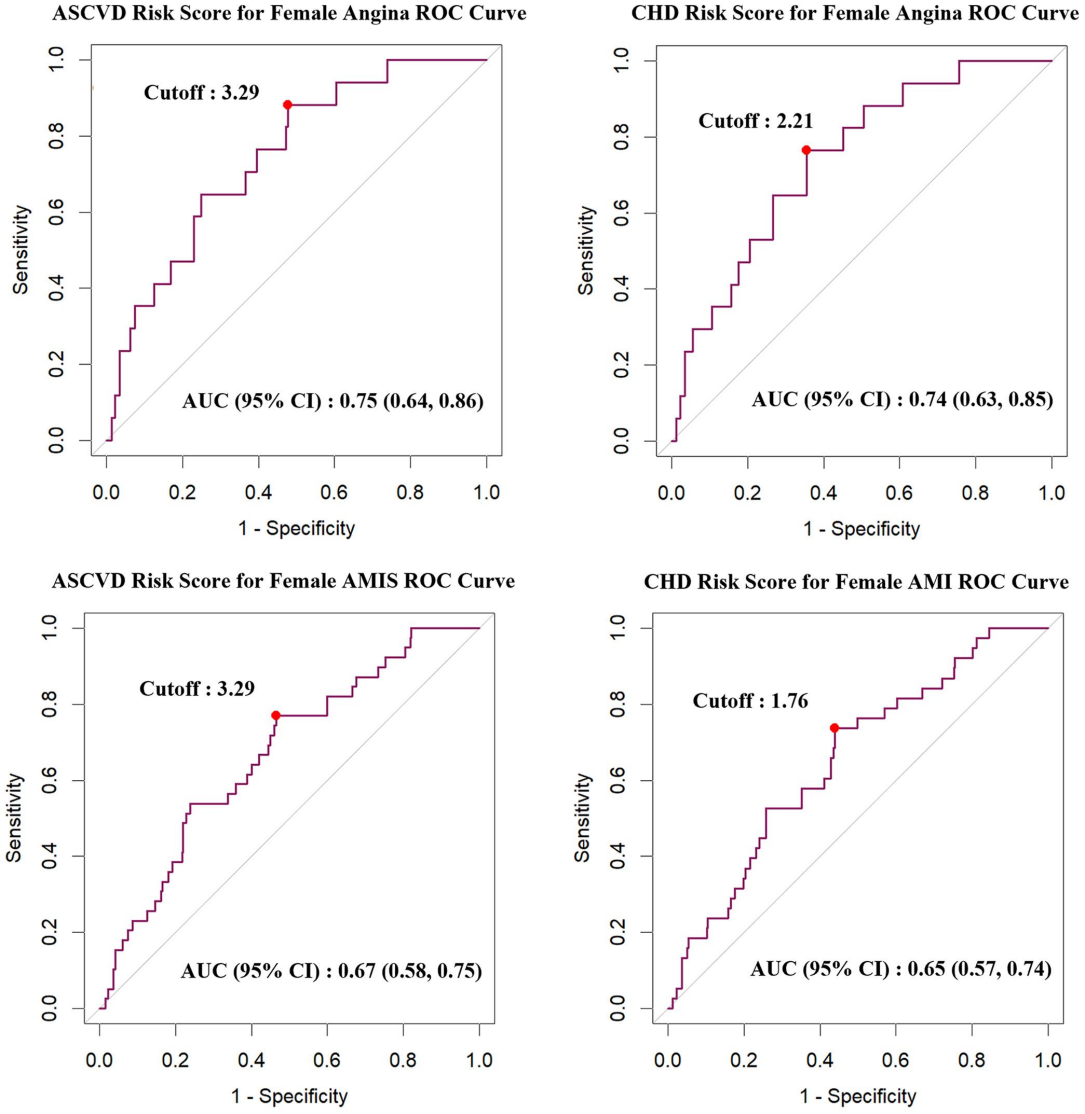
ROC curves of different PREVENT equations risk scores. AMI, angina and myocardial injury; AMIS, angina, myocardial injury and stroke; ASCVD, atherosclerotic cardiovascular disease; CHD, coronary heart disease.

We confirmed the available associations of ASCVD and CHD scores with angina, AMIS, and AMI according to above results. The discrimination was further analyzed. As shown in Figure 4, the ASCVD and CHD risk scores showed a moderate discrimination for postoperative angina, AMIS, and AMI in females: AUC (95% CI) of angina by ASCVD risk score: 0.75 (0.64, 0.86); AUC (95% CI) of angina by CHD risk score: 0.74 (0.63, 0.85); AUC (95% CI) of AMI by CHD risk score: 0.65 (0.57, 0.74); AUC (95% CI) of AMIS by ASCVD risk score: 0.67 (0.58, 0.75).

In the sensitivity analyses performed on the non-imputed dataset, the ASCVD score demonstrated an AUC of 0.74 (95% CI: 0.62–0.86) for angina and 0.68 (95% CI: 0.60–0.77) for AMIS. Similarly, the CHD score achieved an AUC of 0.74 (95% CI: 0.62–0.86) for angina and 0.67 (95% CI: 0.58–0.76) for AMI (Supplementary Figure S6). Besides, sensitivity analyses performed on the dataset with imputation for missing PREVENT equations variables demonstrated similar discriminatory ability. The ASCVD score achieved an AUC of 0.80 (95% CI: 0.74–0.86) for angina and 0.66 (95% CI: 0.60–0.72) for AMIS, and the CHD score yielded an AUC of 0.80 (95% CI: 0.73–0.85) for angina and 0.65 (95% CI: 0.60–0.72) for AMI (Supplementary Figure S7). These results indicated that the discriminative ability of both risk scores were robust.

### The comparations of the PREVENT equations with current predictive models

The AUC of the PREVENT equations for angina, AMIS, and AMI were comparable to those of the RCRI and ThRCRI (all DeLong *P* > 0.05). Furthermore, IDI and NRI analyses showed no additional significant incremental value for the PREVENT equations when compared with either the RCRI nor the ThRCRI (Supplementary Tables S8,S9).

## Discussion

In this retrospective cohort study, we evaluated the sex-specific associations between PREVENT equations and postoperative MACCE in patients undergoing non-cardiac thoracic surgery. In females, both ASCVD and CHD scores were significantly associated with postoperative angina. Furthermore, the ASCVD score was associated with AMIS, and the CHD score with AMI. However, none of the PREVENT equations showed statistically significant associations with any cardiovascular events in males. Through restricted cubic spline and two-stage logistic regression analyses, we identified a nonlinear relationship between the PREVENT HF score and postoperative HF risk in men. This finding parallels the nonlinear association previously reported between the cardiothoracic index (CTI) and cardiovascular events (32). Previous tools, such as the Framingham Risk Score and the PCEs, present several limitations, including limited generalizability beyond white populations, a narrow focus on ASCVD outcomes, and the omission of key predictors like kidney function. The PREVENT equations represent a significant advancement by employing sex-specific, race-free models that integrate eGFR and blood lipids to estimate the risk of total CVD (encompassing both ASCVD and HF) and their subtypes (24, 25). Britton et al. recently validated the PREVENT equations in the NHANES cohort, demonstrating excellent discrimination for CVD-related mortality (C-statistic, 0.890; 95% CI, 0.881–0.898) and superior performance over the PCEs ^(^27). Despite different primary outcomes, our study found that the PREVENT equations provided light to moderate discrimination in a perioperative cohort, particularly for postoperative angina in females assessed by the ASCVD and CHD scores.

The application of the PREVENT equations in Chinese perioperative population extends its clinical applicability. A previous validation of the Framingham CHD Tool in Chinese adults reported AUCs of 0.705 for males and 0.742 for females (33). Our results confirmed that the PREVENT equations maintain light to moderate discriminative capacity in this demographic. However, the existing tools, like the Geriatric-Sensitive Cardiac Risk Index (GSCRI) (8), incorporate clinical outcomes like HF as predictors, may limit their generalizability across populations with varying disease prevalence. For example, in cohorts with high baseline HF prevalence, the GSCRI model might overestimate risk due to reverse causality, whereas in low-prevalence populations, its utility could be compromised. It is precisely this baseline-based approach that allows the PREVENT equations to mitigate the risk of bias from reverse causality and enhances their generalizability across diverse populations. Their performance in non-cardiac thoracic surgical patients underscores the value of clinical associations rooted in universally measurable baseline characteristics. In addition, we compared the AUCs of PREVENT equations with RCRI and ThRCRI, and conducted IDI and NRI analyses. The results showed that there were no significant differences were observed in discriminatory ability between PREVENT equations and RCRI or ThRCRI, and the same is true for IDI and NRI. These results suggest that PREVENT equations possesses discriminatory capabilities similar to these specialized predictive models.

According to the latest 2024 Guidelines for Perioperative Cardiovascular Management in Non-Cardiac Surgery (34), there are some risk calculators that have included traditional risk factors of cardiovascular events, such as age, sex, BMI, current smoking, hypertension medication, diabetes, and so on (4, 10, 11, 13). Dyslipidemia is a known major cardiovascular risk factor. It accelerates atherosclerosis and thereby increases the risk of cardiovascular disease (15–17, 35). The PREVENT equations address this gap by incorporating lipid parameters alongside traditional risk factors. Our findings showed significant association between PREVENT equations and postoperative MACCE, thereby suggesting that the PREVENT equations may be valuable for perioperative risk stratification. However, what must be concerned is that the results may be exploratory. The sensitivity analyses after imputing missing PREVENT equations input variables did not fully validate the robustness. Nevertheless, this sensitivity analysis is merely an exploratory attempt, as imputing core exposure variables will introduce more bias than imputing covariates. More importantly, in dataset with imputation of input variables of PREVENT equations, approximately half of the patients were missing blood lipids. This also exposed the inevitable selection bias of retrospective data.

While we proved a more extensive application of PREVENT equations, several limitations must be concerned. First, the exclusion of over half of the screened participants may have introduced selection bias. Second, the single-center design and relatively small sample size may limit the generalizability of our findings. Future multi-center studies with larger cohorts are needed for robust validation. Third, the potential for residual confounding persists, which may influence the observed associations between the PREVENT equations and outcomes. Forth, a high missing rate of adjusted variables may lead to bias, such as NT-proBNP, PCT and HbA1c. Finally, reliance on electronic medical records may have resulted in an underestimation of postoperative event rates due to incomplete documentation.

## Conclusion

In summary, this study demonstrated a preliminary association between the PREVENT equations and the occurrence of MACCE within 30 days postoperatively in Chinese patients undergoing non-cardiac thoracic surgery. The significant association was mainly focus on females. Further multicenter validation and calibration studies are essential before the PREVENT equations can be incorporated into perioperative risk management strategies.

## Data Availability

The data analyzed in this study is subject to the following licenses/restrictions: The dataset is derived from hospital records and includes confidential patient information. Therefore, it is subject to privacy and ethical restrictions and cannot be made publicly available.Researchers interested in the data may contact the corresponding author or the institutional ethics committee for further information on access restrictions. Requests to access these datasets should be directed to Hua-Min Liu, liuhuamin12@smu.edu.cn.
