# Supplementary material for: Application of predicting risk of cardiovascular disease events equations on postoperative major adverse cardiac and cerebral events for patients undergoing thoracic surgery
Source: Front Cardiovasc Med. 2026 Jul 1;13:1823917. doi: 10.3389/fcvm.2026.1823917 (PMC13369307; doi:10.3389/fcvm.2026.1823917)
Supplement: Supplementary file 1 [file Supplementaryfile1.docx]

**Application of predicting risk of cardiovascular disease events equations on postoperative major adverse cardiac and cerebral events for patients undergoing thoracic surgery**

**Supplement Figures**


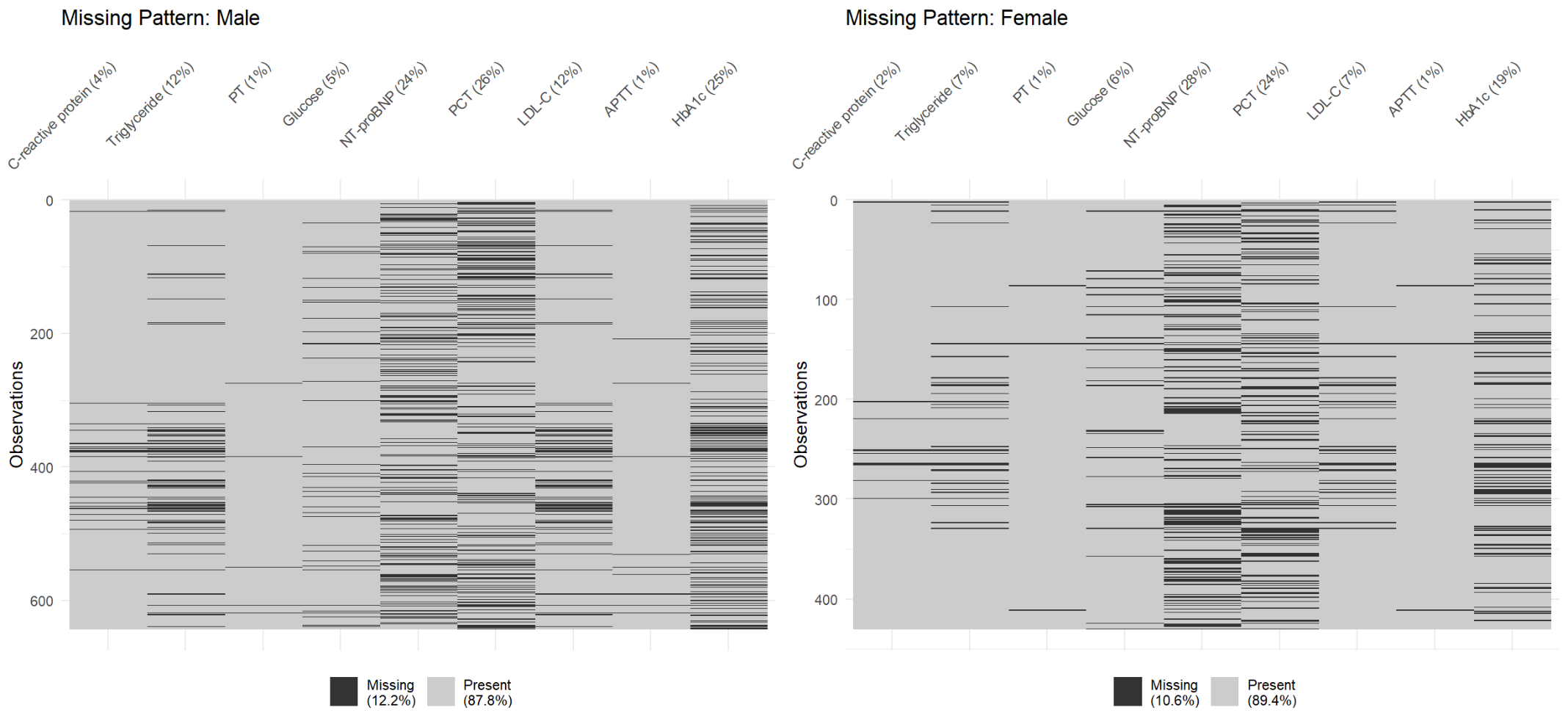


**Figure S1 Heatmap of missing data patterns. Abbreviations:** APTT: activated partial thromboplastin time; NT-proBNP: N-terminal pro-B-type natriuretic peptide; HbA1c: glycated hemoglobin A1c; LDL-C: low-density lipoprotein cholesterol; PCT: plateletcrit; PT: prothrombin time.


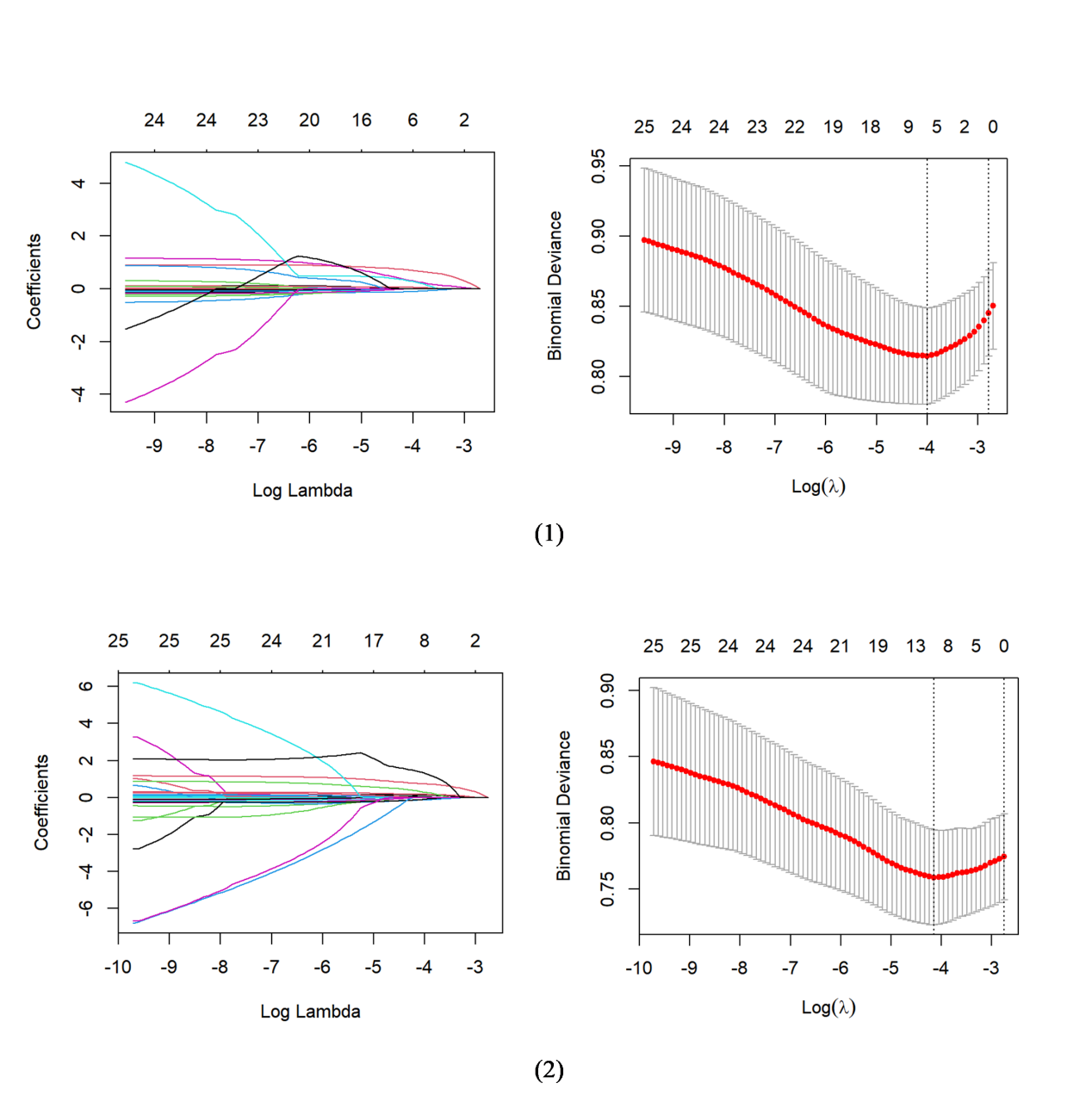
**Figure S2 Variable selection using LASSO regression for males (1) and females (2).**


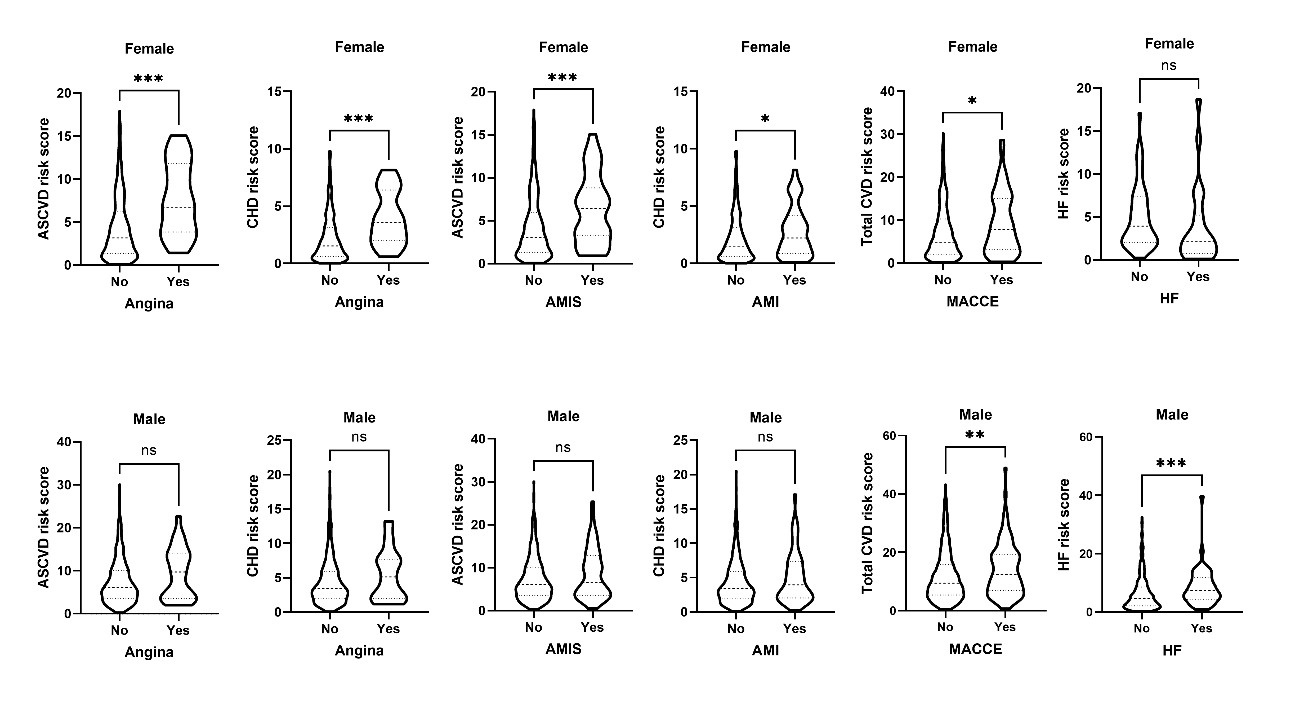


**Figure S3 Violin-plot of PREVENT equations.** ****P* < 0.001; ***P* < 0.01; **P* < 0.05; ns: not significant. **Abbreviations:** AMI, angina and myocardial injury; AMIS, angina, myocardial injury and stroke; ASCVD, atherosclerotic cardiovascular disease; CHD, coronary heart disease; CVD, cardiovascular disease; HF, heart failure; MACCE, major adverse cardiac and cerebral events, including angina, myocardial injury, stroke, or heart failure.


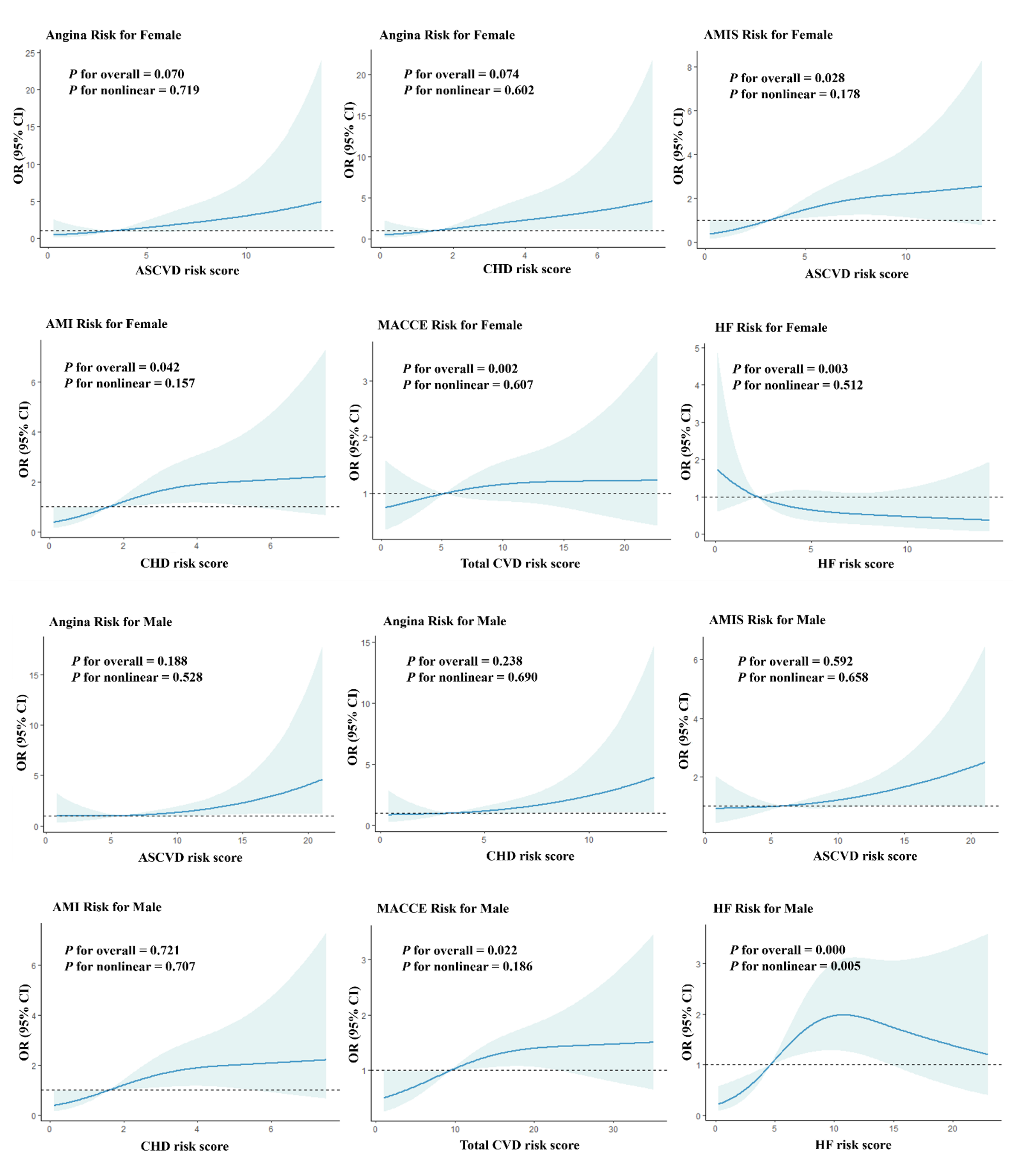


**Figure S4 Restricted cubic spline curve between PREVENT equations and major adverse cardiac and cerebral events using non-imputed dataset.** **Abbreviations:** AMI, angina and myocardial injury; AMIS, angina, myocardial injury and stroke; ASCVD, atherosclerotic cardiovascular disease; CHD, coronary heart disease; CVD, cardiovascular disease; HF, heart failure; MACCE, major adverse cardiac and cerebral events, including angina, myocardial injury, stroke, or heart failure

**
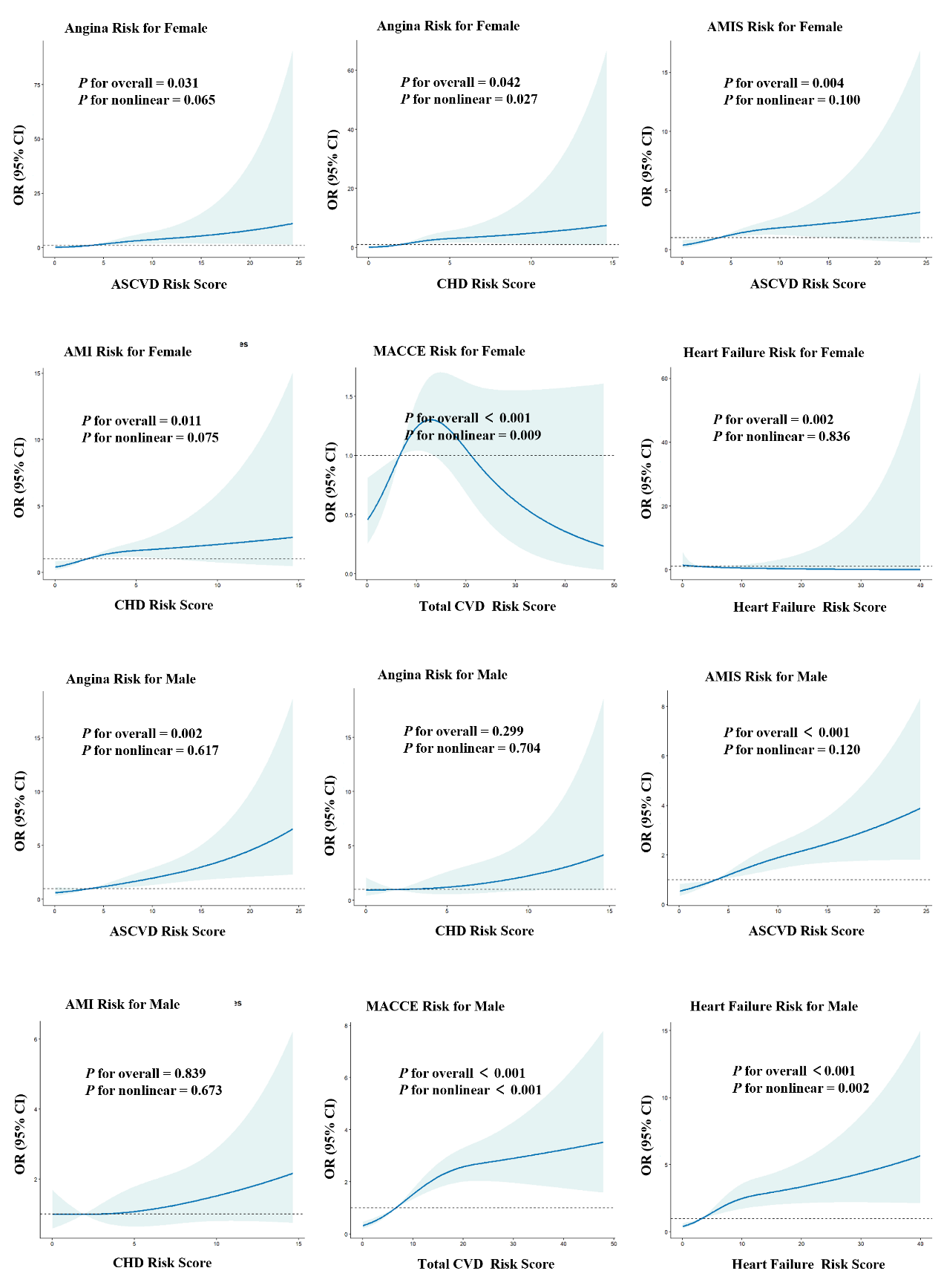
**

**Figure S5 Restricted cubic spline curves** **between PREVENT equations and major adverse cardiac and cerebral events using** **dataset imputed PREVENT input variables.**

**Abbreviations:** AMI, angina and myocardial injury; AMIS, angina, myocardial injury and stroke; ASCVD, atherosclerotic cardiovascular disease; CHD, coronary heart disease; CVD, cardiovascular disease; HF, heart failure; MACCE, major adverse cardiac and cerebral events, including angina, myocardial injury, stroke, or heart failure.

**
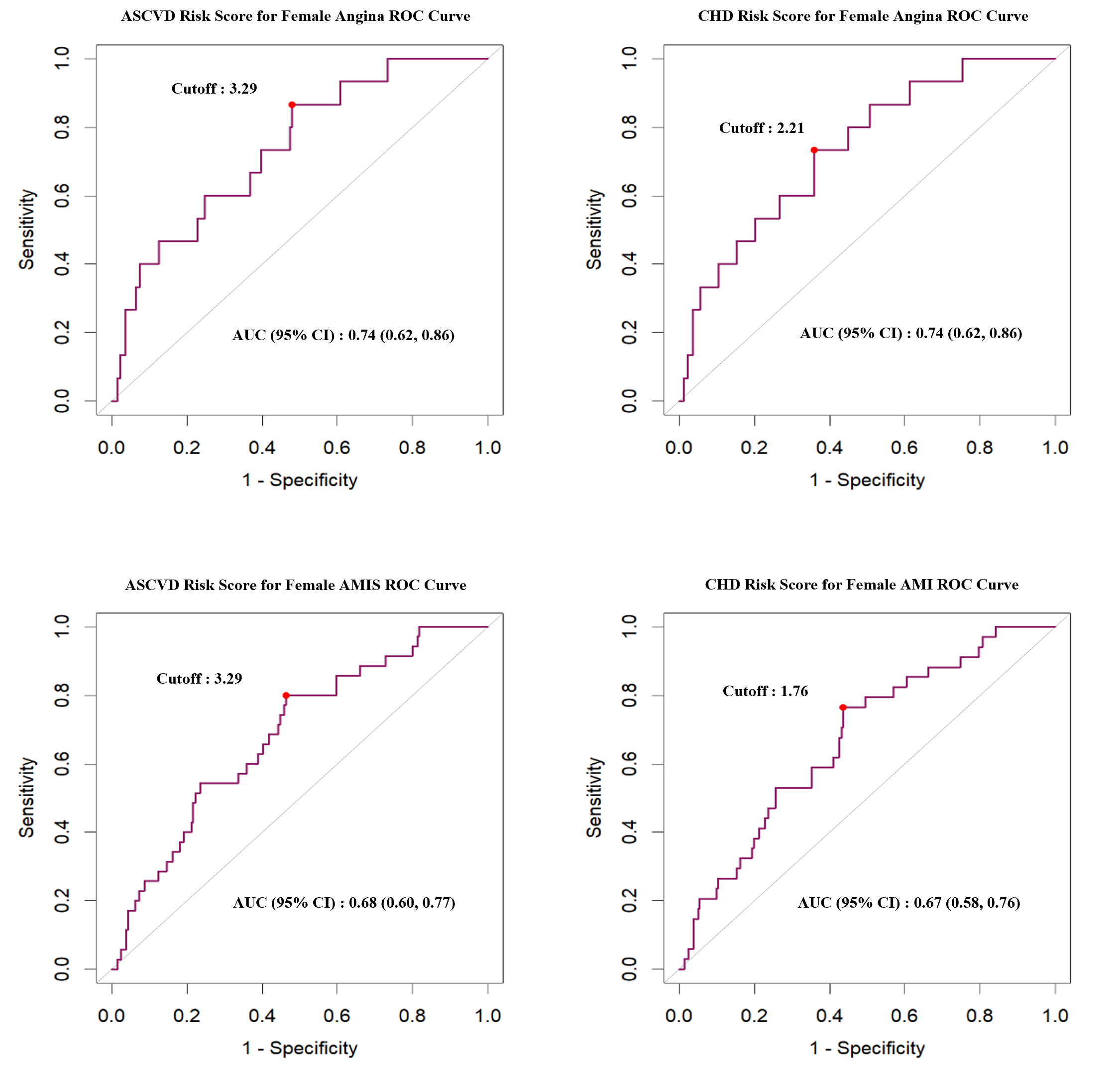
**

**Figure S6 ROC curves between PREVENT equations and major adverse cardiac and cerebral events using non-imputed dataset. Abbreviations:** AMI, angina and myocardial injury; AMIS, angina, myocardial injury and stroke; ASCVD, atherosclerotic cardiovascular disease; CHD, coronary heart disease.

**
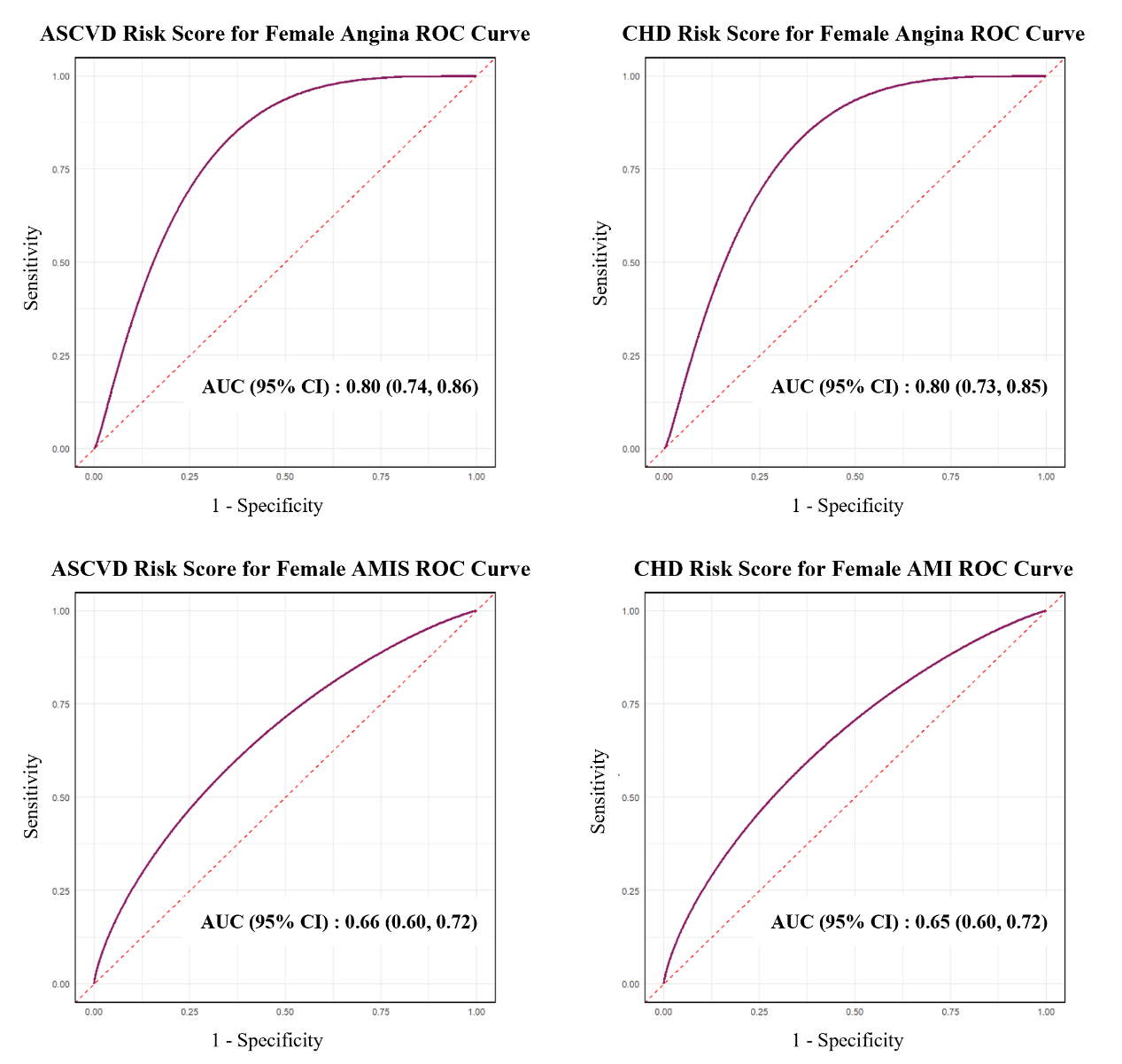
**

**Figure S7 ROC curves between PREVENT equations and major adverse cardiac and cerebral events using** **dataset imputed PREVENT input variables. Abbreviations:** AMI, angina and myocardial injury; AMIS, angina, myocardial injury and stroke; ASCVD, atherosclerotic cardiovascular disease; CHD, coronary heart disease.
