# Supplementary material for: Application of predicting risk of cardiovascular disease events equations on postoperative major adverse cardiac and cerebral events for patients undergoing thoracic surgery
Source: Front Cardiovasc Med. 2026 Jul 1;13:1823917. doi: 10.3389/fcvm.2026.1823917 (PMC13369307; doi:10.3389/fcvm.2026.1823917)
Supplement: Supplementary file 2 [file Supplementaryfile2.docx]

**Application of predicting risk of cardiovascular disease events equations on postoperative major adverse cardiac and cerebral events for patients undergoing thoracic surgery**

**Supplement Tables**

**Table S1 Four PREVENT equations used in this study.**

| PREVENT Model | Female | Male |
| --- | --- | --- |
| Total CVD | -3.307728 +  0.7939329 * (age - 55) / 10 +  0.0305239 * ((Tc - HDL)*0.02586 - 3.5) -  0.1606857 * (HDL*0.02586 - 1.3) / 0.3 -  0.2394003 * (pmin (SBP, 110) - 110) / 20 +  0.360078 * (pmax (SBP, 110) - 130) / 20 +  0.8667604 * diabetes +  0.5360739 * current_smoker +  0.6045917 * (pmin (eGFR, 60) - 60) / -15 +  0.0433769 * (pmax (eGFR, 60) - 90) / -15 +  0.3151672 * anti_hypertensive_med -  0.1477655 * statin -  0.0663612 * anti_hypertensive_med * (pmax (SBP, 110) - 130) / 20 +  0.1197879 * statin * ((Tc - HDL)*0.02586 - 3.5) -  0.0819715 * (age - 55) / 10 * ((Tc - HDL)*0.02586 - 3.5) +  0.0306769 * (age - 55) / 10 * (HDL*0.02586 - 1.3) / 0.3 -  0.0946348 * (age - 55) / 10 * (pmax (SBP, 110) - 130) / 20 -  0.27057 * (age - 55) / 10 * diabetes -  0.078715 * (age - 55) / 10 * current_smoker -  0.1637806 * (age - 55) / 10 * (pmin (eGFR, 60) - 60) / -15 | -3.031168 +  0.7688528 * (age - 55) / 10 +  0.0736174 * ((Tc - HDL)*0.02586 - 3.5) -  0.0954431 * (HDL*0.02586 - 1.3) / 0.3 -  0.4347345 * (pmin (SBP, 110) - 110) / 20 +  0.3362658 * (pmax (SBP, 110) - 130) / 20 +  0.7692857 * diabetes +  0.4386871 * current_smoker +  0.5378979 * (pmin (eGFR, 60) - 60) / -15 +  0.0164827 * (pmax (eGFR, 60) - 90) / -15 +  0.288879 * anti_hypertensive_med -  0.1337349 * statin -  0.0475924 * anti_hypertensive_med * (pmax (SBP, 110) - 130) / 20 +  0.150273 * statin * ((Tc - HDL)*0.02586 - 3.5) -  0.0517874 * (age - 55) / 10 * ((Tc - HDL)*0.02586 - 3.5) +  0.0191169 * (age - 55) / 10 * (HDL*0.02586 - 1.3) / 0.3 -  0.1049477 * (age - 55) / 10 * (pmax (SBP, 110) - 130) / 20 -  0.2251948 * (age - 55) / 10 * diabetes -  0.0895067 * (age - 55) / 10 * current_smoker -  0.1543702 * (age - 55) / 10 * (pmin (eGFR, 60) - 60) / -15 |
| ASCVD | -3.819975 +  0.719883 * (age - 55) / 10 +  0.1176967 * ((Tc - HDL)*0.02586 - 3.5) -  0.151185 * (HDL*0.02586 - 1.3) / 0.3 -  0.0835358 * (pmin (SBP, 110) - 110) / 20 +  0.3592852 * (pmax (SBP, 110) - 130) / 20 +  0.8348585 * diabetes +  0.4831078 * current_smoker +  0.4864619 * (pmin (eGFR, 60) - 60) / -15 +  0.0397779 * (pmax (eGFR, 60) - 90) / -15 +  0.2265309 * anti_hypertensive_med -  0.0592374 * statin -  0.0395762 * anti_hypertensive_med * (pmax (SBP, 110) - 130) / 20 +  0.0844423 * statin * ((Tc - HDL)*0.02586 - 3.5) -  0.0567839 * (age - 55) / 10 * ((Tc - HDL)*0.02586 - 3.5) +  0.0325692 * (age - 55) / 10 * (HDL*0.02586 - 1.3) / 0.3 -  0.1035985 * (age - 55) / 10 * (pmax (SBP, 110) - 130) / 20 -  0.2417542 * (age - 55) / 10 * diabetes -  0.0791142 * (age - 55) / 10 * current_smoker -  0.1671492 * (age - 55) / 10 * (pmin (eGFR, 60) - 60) / -15 | -3.500655 +  0.7099847 * (age - 55) / 10 +  0.1658663 * ((Tc - HDL)*0.02586 - 3.5) -  0.1144285 * (HDL*0.02586 - 1.3) / 0.3 -  0.2837212 * (pmin (SBP, 110) - 110) / 20 +  0.3239977 * (pmax (SBP, 110) - 130) / 20 +  0.7189597 * diabetes +  0.3956973 * current_smoker +  0.3690075 * (pmin (eGFR, 60) - 60) / -15 +  0.0203619 * (pmax (eGFR, 60) - 90) / -15 +  0.2036522 * anti_hypertensive_med -  0.0865581 * statin -  0.0322916 * anti_hypertensive_med * (pmax (SBP, 110) - 130) / 20 +  0.114563 * statin * ((Tc - HDL)*0.02586 - 3.5) -  0.0300005 * (age - 55) / 10 * ((Tc - HDL)*0.02586 - 3.5) +  0.0232747 * (age - 55) / 10 * (HDL*0.02586 - 1.3) / 0.3 -  0.0927024 * (age - 55) / 10 * (pmax (SBP, 110) - 130) / 20 -  0.2018525 * (age - 55) / 10 * diabetes -  0.0970527 * (age - 55) / 10 * current_smoker -  0.1217081 * (age - 55) / 10 * (pmin (eGFR, 60) - 60) / -15 |
| HF | -4.310409 +  0.8998235 * (age - 55) / 10 -  0.4559771 * (pmin (SBP, 110) - 110) / 20 +  0.3576505 * (pmax (SBP, 110) - 130) / 20 +  1.038346 * diabetes +  0.583916 * current_smoker -  0.0072294 * (pmin (BMI, 30) - 25) / 5 +  0.2997706 * (pmax (BMI, 30) - 30) / 5 +  0.7451638 * (pmin (eGFR, 60) - 60) / -15 +  0.0557087 * (pmax (eGFR, 60) - 90) / -15 +  0.3534442 * anti_hypertensive_med -  0.0981511 * anti_hypertensive_med * (pmax (SBP, 110) - 130) / 20 -  0.0946663 * (age - 55) / 10 * (pmax (SBP, 110) - 130) / 20 -  0.3581041 * (age - 55) / 10 * diabetes -  0.1159453 * (age - 55) / 10 * current_smoker -  0.003878 * (pmax (BMI, 30) - 30) / 5 -  0.1884289 * (age - 55) / 10 * (pmin (eGFR, 60) - 60) / -15 | - 3.946391 +  0.8972642 * (age - 55) / 10 -  0.6811466 * (pmin (SBP, 110) - 110) / 20 +  0.3634461 * (pmax (SBP, 110) - 130) / 20 +  0.923776 * diabetes +  0.5023736 * current_smoker -  0.0485841 * (pmin (BMI, 30) - 25) / 5 +  0.3726929 * (pmax (BMI, 30) - 30) / 5 +  0.6926917 * (pmin (eGFR, 60) - 60) / -15 +  0.0251827 * (pmax (eGFR, 60) - 90) / -15 +  0.2980922 * anti_hypertensive_med -  0.0497731 * anti_hypertensive_med * (pmax (SBP, 110) - 130) / 20 -  0.1289201 * (age - 55) / 10 * (  - 130) / 20 -  0.3040924 * (age - 55) / 10 * diabetes -  0.1401688 * (age - 55) / 10 * current_smoker -  0.0068126 * (pmax (BMI, 30) - 30) / 5 -  0.1797778 * (age - 55) / 10 * (pmin (eGFR, 60) - 60) / -15 |
| CHD | -4.608751 +  0.7587146 * (age - 55) / 10 +  0.1810949 * ((Tc - HDL)*0.02586 - 3.5) -  0.2014507 * (HDL*0.02586 - 1.3) / 0.3 -  0.0881827 * (pmin (SBP, 110) - 110) / 20 +  0.3547731 * (pmax (SBP, 110) - 130) / 20 +  0.9045358 * diabetes +  0.5410917 * current_smoker +  0.5198725 * (pmin (eGFR, 60) - 60) / -15 +  0.0325935 * (pmax (eGFR, 60) - 90) / -15 +  0.2010642 * anti_hypertensive_med -  0.036195 * statin -  0.0891238 * anti_hypertensive_med * (pmax (SBP, 110) - 130) / 20 +  0.0750716 * statin * ((Tc - HDL)*0.02586 - 3.5) -  0.0683256 * (age - 55) / 10 * ((Tc - HDL)*0.02586 - 3.5) +  0.0484755 * (age - 55) / 10 * (HDL*0.02586 - 1.3) / 0.3 -  0.0898086 * (age - 55) / 10 * (pmax (SBP, 110) - 130) / 20 -  0.2569041 * (age - 55) / 10 * diabetes -  0.0786607 * (age - 55) / 10 * current_smoker -  0.1597513 * (age - 55) / 10 * (pmin (eGFR, 60) - 60) / -15 | -4.156753 +  0.7423283 * (age - 55) / 10 +  0.2572109 * ((Tc - HDL)*0.02586 - 3.5) -  0.1820374 * (HDL*0.02586 - 1.3) / 0.3 -  0.3174515 * (pmin (SBP, 110) - 110) / 20 +  0.312778 * (pmax (SBP, 110) - 130) / 20 +  0.7485249 * diabetes +  0.3912047 * current_smoker +  0.376487 * (pmin (eGFR, 60) - 60) / -15 +  0.0193687 * (pmax (eGFR, 60) - 90) / -15 +  0.1588199 * anti_hypertensive_med -  0.0494555 * statin -  0.0577851 * anti_hypertensive_med * (pmax (SBP, 110) - 130) / 20 +  0.0809765 * statin * ((Tc - HDL)*0.02586 - 3.5) -  0.0517872 * (age - 55) / 10 * ((Tc - HDL)*0.02586 - 3.5) +  0.0489033 * (age - 55) / 10 * (HDL*0.02586 - 1.3) / 0.3 -  0.0850404 * (age - 55) / 10 * (pmax (SBP, 110) - 130) / 20 -  0.2107552 * (age - 55) / 10 * diabetes -  0.1206397 * (age - 55) / 10 * current_smoker -  0.07795 * (age - 55) / 10 * (pmin (eGFR, 60) - 60) / -15 |

**Abbreviations:** ASCVD, atherosclerotic cardiovascular disease. BMI, body mass index; CHD, coronary heart disease; CVD, cardiovascular disease; eGFR, estimated glomerular filtration Rate; HDL, high-density lipoprotein; HF, heart failure; PREVENT, predicting risk of cardiovascular disease events; SBP, systolic blood pressure; TC, total cholesterol.

**Table S2 Proportions of missing data for covariates in 1073 included patients.**

| Variables | Male missing cases | Male missing rate | Female missing cases | Female missing rate |
| --- | --- | --- | --- | --- |
| hemoglobin | 0 | 0% | 0 | 0% |
| platelets | 0 | 0% | 0 | 0% |
| white blood cell count | 0 | 0% | 0 | 0% |
| absolute neutrophil count | 0 | 0% | 0 | 0% |
| neutrophil (%) | 0 | 0% | 0 | 0% |
| absolute lymphocyte count | 0 | 0% | 0 | 0% |
| lymphocyte (%) | 0 | 0% | 0 | 0% |
| C-reactive protein (CRP) | 26 | 4% | 9 | 2% |
| procalcitonin (PCT) | 167 | 26% | 103 | 24% |
| triglycerides (TG) | 77 | 12% | 30 | 7% |
| low-density lipoprotein (LDL) | 77 | 12% | 30 | 7% |
| albumin | 0 | 0% | 0 | 0% |
| prothrombin time (PT) | 6 | 1% | 4 | 1% |
| activated partial thromboplastin time (APTT) | 6 | 1% | 4 | 1% |
| glycated hemoglobin (HbA1c) | 161 | 25% | 82 | 19% |
| glucose | 32 | 5% | 26 | 6% |
| N-terminal pro-B-type natriuretic peptide (NT-proBNP) | 154 | 24% | 120 | 28% |
| atrial arrhythmia | 0 | 0% | 0 | 0% |
| arrhythmia | 0 | 0% | 0 | 0% |
| aspirin use | 0 | 0% | 0 | 0% |
| current drinking | 0 | 0% | 0 | 0% |
| American Society of Anesthesiologists (ASA) Physical Status | 0 | 0% | 0 | 0% |

**Table S****3 Logistic regression for the association between HF scores and HF risk in males (Take 10.998 as the binary classification point) *.**

| **HF scores** | **OR (95% CI) of HF** | | |
| --- | --- | --- | --- |
|  | **Model 1** | **Model 2** | **Model 3** |
|  | **OR (95% CI)** | **OR (95% CI)** | **OR (95% CI)** |
| HF score > 10.998 | 0.72 (0.34, 1.53) | 0.74 (0.35, 1.58) | 0.57 (0.26, 1.28) |
| HF score ≤ 10.998 | 2.55 (1.30, 5.00) | 2.86 (1.41,5.79) | 2.55 (1.23, 5.28) |

**Abbreviations:** HF, heart failure; Model 1: unadjusted; Model 2: adjusted for atrial arrhythmia, aspirin, and current-drink; Model 3: adjusted for atrial arrhythmia, aspirin, current-drink, C-reactive protein (CRP), hemoglobin, and triglyceride. *This is a validation test based on our RCS results; thus, the correction of multiple comparison was not performed.

**Table S4 Sensitivity analyses for the association between PREVENT equations and major adverse cardiac and cerebral events in dataset without imputation.**

| **Risk score*** | **Outcome** | **OR (95% CI)** | **P value** | **FDR corrected**  ***P* values** |
| --- | --- | --- | --- | --- |
| **Females^§^** |  |  |  |  |
| ASCVD | AMIS | 1.62 (1.14, 2.29) | 0.008 | 0.047 |
| ASCVD | Angina | 1.91 (1.11, 3.28) | 0.026 | 0.051 |
| CHD | Angina | 1.84 (1.09, 3.10) | 0.029 | 0.043 |
| CHD | AMI | 1.52 (1.08, 2.14) | 0.019 | 0.056 |
| HF | HF | 0.68 (0.42, 1.11) | 0.120 | 0.144 |
| Total CVD | MACCE | 1.13 (0.84, 1.54) | 0.419 | 0.419 |
| **Males^£^** |  |  |  |  |
| ASCVD | AMIS | 1.29 (0.99, 1.66) | 0.057 | 0.069 |
| ASCVD | Angina | 1.52 (1.03, 2.24) | 0.034 | 0.068 |
| CHD | Angina | 1.48 (1.01, 2.16) | 0.044 | 0.066 |
| CHD | AMI | 1.24 (0.96, 1.61) | 0.103 | 0.103 |
| HF | HF | 1.39 (1.06, 1.82) | 0.018 | 0.053 |
| Total CVD | MACCE | 1.32 (1.06, 1.63) | 0.011 | 0.068 |

*Risk scores were Z-transformed. **^§^**In females, adjusted for ASA classification, arrhythmia, aspirin, current-drink, hemoglobin, neutrophils (%), C-reactive protein (CRP), Prothrombin Time (PT), glucose, and N-terminal pro-B-type Natriuretic Peptide (NT-proBNP). **^£^**In males, adjusted for atrial arrhythmia, aspirin, current-drink, hemoglobin, CRP, and triglyceride.

**Table S5 Comparisons of baseline characteristics between the included patients and the patients who lack any input of PREVENT equations.**

| **Characteristics** | **Included cohort**  **(n=1073)** | **Excluded cohort^‡^**  **(n=1408)** | ***P* value** |
| --- | --- | --- | --- |
| **Male (n)** | 643 | 784 |  |
| Age (years), Median (IQR) | 58 (52-66) | 54 (43-64) | <0.001 |
| BMI (kg/m²), Median (IQR) | 23.5 (21.6-25.8) | 22.9 (20.0-25.1) | <0.001 |
| Diabetes, n (%) | 127 (19.7) | 91 (11.6) | <0.001 |
| Hypertension medication, n (%) | 219 (34.0) | 154 (19.6) | <0.001 |
| Hemoglobin (g/L), Median (IQR) | 138.0 (126.0-147.0) | 139.0 (126.0-149.0) | <0.001 |
| CRP (mg/L), Median (IQR) | 1.7 (0.7-4.3) | 2.2 (0.7-8.0) | 0.002 |
| Triglyceride (mg/dL), Median (IQR) | 118.0 (86.0-181.0) | 104.2 (81.1-124.2) | <0.001 |
| Neutrophil (%), Median (IQR) | 58.4 (53.0-65.0) | 58.1 (51.9-65.1) | 0.254 |
| PT (seconds), Median (IQR) | 17.7 (17.0-18.4) | 17.7 (17.0-18.4) | 0.775 |
| Glucose (mmol/L), Median (IQR) | 5.1 (4.6-5.7) | 5.0 (4.6-5.5) | 0.008 |
| NT-proBNP (pg/mL), Median (IQR) | 38.5 (20.7-71.1) | 41.9 (20.8-101.8) | 0.061 |
| Atrial arrhythmia, n (%) | 28 (4.4) | 12 (1.5) | 0.002 |
| Aspirin medication, n (%) | 38 (5.9) | 15 (1.9) | <0.001 |
| Current Drink, n (%) | 292 (45.4) | 283 (36.1) | <0.001 |
| Arrhythmia, n (%) | 29 (4.5) | 15 (1.9) | 0.008 |
| ASA, n (%) |  |  | <0.001 |
| Ⅰ | 25 (3.9) | 98 (12.5) |  |
| Ⅱ | 518 (80.6) | 577 (73.6) |  |
| ≥Ⅲ | 100 (15.6) | 109 (13.9) |  |
| HF | 48 (7.5%) | 52 (6.6%) | 0.611 |
| Angina | 25 (3.9%) | 33 (4.2%) | 0.864 |
| AMI | 58 (9.0%) | 76 (9.7%) | 0.732 |
| AMIS | 59 (9.2%) | 77 (9.8%) | 0.747 |
| MACCE, n (%) | 99 (15.4) | 115 (14.7) | 0.701 |
| **Female (n)** | 430 | 624 |  |
| Age (years), Median (IQR) | 58 (50-65) | 53 (43-61) | <0.001 |
| BMI (kg/m²), Median (IQR) | 23.1 (21.2-25.2) | 22.3 (20.2-24.7) | <0.001 |
| Diabetes, n (%) | 72 (16.7) | 61 (9.8) | 0.001 |
| Hypertension medication, n (%) | 149 (34.6) | 124 (19.9) | <0.001 |
| Hemoglobin (g/L), Median (IQR) | 124 (116-132) | 124 (115-131) | 0.099 |
| CRP (mg/L), Median (IQR) | 1.4 (0.7-3.8) | 1.1 (0.5-2.4) | <0.001 |
| Triglyceride (mg/dL), Median (IQR) | 125.0 (92.3-177.8) | 97.9 (83.7-126.5) | <0.001 |
| Neutrophil (%), Median (IQR) | 57.5 (50.9-64.7) | 56.2 (49.9-62.1) | 0.011 |
| PT (seconds), Median (IQR) | 17.7 (17.0-18.4) | 17.8 (17.2-18.4) | 0.211 |
| Glucose (mmol/L), Median (IQR) | 5.0 (4.7-5.5) | 4.9 (4.5-5.3) | <0.001 |
| NT-proBNP (pg/mL), Median (IQR) | 42.0 (24.2-77.4) | 51.04 (26.8-105.3) | 0.026 |
| Atrial arrhythmia, n (%) | 8 (1.9) | 8 (1.3) | 0.618 |
| Aspirin medication, n (%) | 31 (7.2) | 10 (1.6) | <0.001 |
| Current Drink, n (%) | 52 (12.1) | 63 (10.1) | 0.357 |
| Arrhythmia, n (%) | 9 (2.1) | 8 (1.3) | 0.436 |
| ASA, n (%) |  |  | <0.001 |
| Ⅰ | 16 (3.7) | 78 (12.5) |  |
| Ⅱ | 359 (83.5) | 499 (80.0) |  |
| ≥Ⅲ | 55 (12.8) | 47 (7.5) |  |
| HF | 23 (5.3%) | 31 (5.0%) | 0.894 |
| Angina | 17 (4.0%) | 17 (2.7%) | 0.351 |
| AMI | 38 (8.8%) | 49 (7.9%) | 0.648 |
| AMIS | 39 (9.1%) | 49 (7.9%) | 0.556 |
| MACCE | 56(13.0) | 82 (13.1%) | 0.955 |

^‡^Excluded due to the lack of any input of PREVENT equations. **Abbreviations:** AMI, angina and myocardial injury; AMIS, angina, myocardial injury and stroke; ASCVD, atherosclerotic cardiovascular disease; ASA, American Society of Anesthesiologists physical status classification; BMI, Body Mass Index; CHD, coronary heart disease; CRP, C-reactive protein; CVD, cardiovascular disease; HF, heart failure; IQR, Interquartile Range; MACCE, major adverse cardiac and cerebral events, including angina, myocardial injury, stroke, or heart failure; NT-proBNP, N-terminal pro-B-type Natriuretic Peptide; PT, Prothrombin Time.

**Table S6 Proportions of missing data for PREVENT equations input variables.**

| **Variables** | Male missing cases | Male missing rate | Female missing cases | Female missing rate |
| --- | --- | --- | --- | --- |
| Tc | 652 | 45.7% | 528 | 50.1% |
| HDL | 652 | 45.7% | 528 | 50.1% |
| SBP | 56 | 3.9% | 16 | 1.5% |
| BMI | 9 | 0.6% | 4 | 0.4% |
| eGFR | 2 | 0.1% | 4 | 0.4% |
| Age | 0 | 0% | 0 | 0% |
| Current_smoker | 0 | 0% | 0 | 0% |
| Diabetes | 0 | 0% | 0 | 0% |
| Statin | 0 | 0% | 0 | 0% |
| Anti_hypertensive_med | 0 | 0% | 0 | 0% |

**Abbreviations:** Anti_hypertensive_med, antihypertensive medication; BMI, body mass index; eGFR, estimated glomerular filtration rate; HDL, high-density lipoprotein cholesterol; SBP, systolic blood pressure; Tc, total cholesterol.

**Table S7 Sensitivity analyses by imputing PREVENT equations inputs for the associations between PREVENT equations and major adverse cardiac and cerebral events (n = 2481).**

| **Risk score*** | **Outcome** | **OR (95% CI)** | **P value** | **FDR corrected**  ***P* values** |
| --- | --- | --- | --- | --- |
| **Females^§^** |  |  |  |  |
| ASCVD | AMIS | (1.22–2.24) | 0.001 | 0.003 |
| ASCVD | Angina | (1.45–3.39) | <0.001 | <0.001 |
| CHD | Angina | (1.33–3.09) | 0.001 | 0.002 |
| CHD | AMI | (1.15–2.14) | 0.005 | 0.0075 |
| HF | HF | (0.24–1.22) | 0.196 | 0.196 |
| Total CVD | MACCE | (1.05–1.71) | 0.021 | 0.0252 |
| **Males^£^** |  |  |  |  |
| ASCVD | AMIS | (1.26–1.71) | <0.001 | <0.001 |
| ASCVD | Angina | (1.28–1.97) | <0.001 | <0.001 |
| CHD | Angina | (0.96–1.85) | 0.073 | 0.0876 |
| CHD | AMI | (0.92–1.46) | 0.178 | 0.178 |
| HF | HF | (1.26–1.72) | <0.001 | <0.001 |
| Total CVD | MACCE | (1.30–1.69) | <0.001 | <0.001 |

*Risk scores were Z-transformed. **^§^**Adjusted for ASA classification, arrhythmia, aspirin, current-drink, hemoglobin, neutrophils (%), C-reactive protein (CRP), PT, glucose, and NT-proBNP. **^£^**Adjusted for atrial arrhythmia, aspirin, current-drink, hemoglobin, CRP, and triglyceride.

**Table S8. Comparisons of the PREVENT equations with RCRI in females.**

| **Outcomes** | **PREVENT score** | **AUC for RCRI** | **AUC for PREVENT** | **Delta AUC** | **DeLong *P*** | **IDI** | **IDI 95% CI** | **IDI *P*** | **NRI** | **NRI 95% CI** | **NRI *P*** |
| --- | --- | --- | --- | --- | --- | --- | --- | --- | --- | --- | --- |
| Angina | ASCVD | 0.702 | 0.747 | 0.045 | 0.473 | -0.013 | (-0.043, 0.018) | 0.408 | 0.005 | (-0.480, 0.519) | 0.984 |
| AMIS | ASCVD | 0.625 | 0.669 | 0.043 | 0.330 | -0.040 | (-0.114, 0.014) | 0.226 | 0.079 | (-0.260, 0.425) | 0.642 |
| Angina | CHD | 0.702 | 0.743 | 0.041 | 0.515 | -0.016 | (-0.047, 0.015) | 0.311 | 0.123 | (-0.395, 0.638) | 0.641 |
| AMI | CHD | 0.628 | 0.654 | 0.026 | 0.561 | -0.049 | (-0.128, 0.007) | 0.158 | 0.046 | (-0.286, 0.400) | 0.788 |

**Abbreviations:** AMI, Acute Myocardial Infarction; AMIS, Angina, Myocardial Infarction and Stroke; ASCVD, Atherosclerotic Cardiovascular Disease; AUC, Area Under the Curve; CHD, Coronary Heart Disease; CI, Confidence Interval; IDI, Integrated Discrimination Improvement; NRI, Net Reclassification Improvement; PREVENT, Predicting Risk of Vascular Events in Non-cardiac Surgery Patients; RCRI, Revised Cardiac Risk Index.

**Table S9. Comparison of the PREVENT equations with ThRCRI in females.**

| **Outcomes** | **PREVENT score** | **AUC for ThRCRI** | **AUC for PREVENT** | **Delta AUC** | **DeLong *P*** | **IDI** | **IDI 95% CI** | **IDI *P*** | **NRI** | **NRI 95% CI** | **NRI *P*** |
| --- | --- | --- | --- | --- | --- | --- | --- | --- | --- | --- | --- |
| Angina | ASCVD | 0.702 | 0.747 | 0.045 | 0.473 | -0.013 | (-0.043–0.018) | 0.408 | 0.005 | (-0.480–0.519) | 0.984 |
| AMIS | ASCVD | 0.625 | 0.669 | 0.043 | 0.330 | -0.040 | (-0.114–0.014) | 0.226 | 0.079 | (-0.260–0.425) | 0.642 |
| Angina | CHD | 0.702 | 0.743 | 0.041 | 0.515 | -0.016 | (-0.047–0.015) | 0.311 | 0.123 | (-0.395–0.638) | 0.641 |
| AMI | CHD | 0.628 | 0.655 | 0.027 | 0.561 | -0.049 | (-0.128–0.007) | 0.158 | 0.046 | (-0.286–0.400) | 0.788 |

**Abbreviations:** AMI, Acute Myocardial Infarction; AMIS, Angina, Myocardial Infarction and Stroke; ASCVD, Atherosclerotic Cardiovascular Disease; AUC, Area Under the Curve; CHD, Coronary Heart Disease; CI, Confidence Interval; IDI, Integrated Discrimination Improvement; NRI, Net Reclassification Improvement; PREVENT, Predicting Risk of Vascular Events in Non-cardiac Surgery Patients; ThRCRI, Thoracic Revised Cardiac Risk Index.
